# Nonunion in Long Bone Fractures: A Comprehensive Review of Current Treatment Strategies

**DOI:** 10.7759/cureus.97599

**Published:** 2025-11-23

**Authors:** Ahmed Mohamed, Daniel Francis, Usman Fuad, Nabil Elmaleh, Ahmed Nagi

**Affiliations:** 1 Trauma and Orthopaedics, Royal Cornwall Hospital, Truro, GBR; 2 Trauma and Orthopaedics, University Hospitals of Morecambe Bay NHS Foundation Trust, Lancaster, GBR; 3 Trauma and Orthopaedics, University Hospitals Derby and Burton NHS Foundation Trust, Derby, GBR

**Keywords:** bone grafting, bone morphogenetic protein, fracture healing, internal fixation, long bone fractures, nonunion, orthopedic surgery

## Abstract

Nonunion is a significant complication of long bone fractures, occurring when bone healing fails to progress despite adequate time and appropriate initial management. This condition affects a significant proportion of fractures, with rates varying considerably based on the anatomical location, fracture pattern, patient factors, and treatment methods. The management of nonunion remains challenging and requires a comprehensive understanding of the mechanical and biological factors influencing bone healing. This narrative review systematically examines the current treatment options available for managing nonunion in long bone fractures, including both nonoperative and operative approaches. Nonoperative management encompasses the optimization of patient factors, pharmacological interventions, and biophysical stimulation techniques. Operative treatment strategies include revision internal fixation, bone grafting, biological augmentation with growth factors, distraction osteogenesis, and vascularized bone transfer. The selection of an appropriate treatment modality depends on multiple factors, including the type of nonunion, anatomical location, presence of infection, bone quality, soft-tissue condition, and patient comorbidities. Recent advances in surgical techniques, biological augmentation, and fixation devices have improved outcomes; however, successful management fundamentally requires restoration of mechanical stability combined with enhancement of the biological environment. This review synthesizes the current evidence regarding various treatment modalities, their indications, techniques, outcomes, and complications, providing a practical framework for clinicians managing this complex orthopedic problem.

## Introduction and background

Nonunion is one of the most challenging complications of orthopedic trauma surgery. Although bone possesses remarkable regenerative capacity, certain fractures fail to heal despite appropriate initial treatment and adequate time for healing [1]. The reported incidence of nonunion ranges from 5% to 10% overall but can reach significantly higher rates in specific fracture types and locations. For instance, scaphoid fractures demonstrate nonunion rates approaching 12-15%, while distal femoral fractures may exhibit rates exceeding 20% in complex cases [2].

Nonunion affects patients' quality of life, functional capacity, and socioeconomic and psychological status. From a healthcare perspective, nonunion management represents a substantial economic burden, requiring multiple interventions, prolonged treatment courses, and extensive rehabilitation [3].

Historically, the understanding and management of nonunions have evolved. Early practitioners recognized that some fractures failed to heal, but lacked effective treatment options. The pioneering work of surgeons in the 19th and 20th centuries gradually established the principles of fracture fixation and bone grafting that form the foundation of modern nonunion management. The development of internal fixation devices, bone grafting techniques, and, more recently, biological augmentation strategies has dramatically improved outcomes for patients with nonunion [4,5].

Despite these advances, nonunion management remains complex and challenging. When operative intervention is indicated, success requires a thorough understanding of the underlying pathophysiology, careful patient assessment, appropriate treatment selection, and meticulous surgical techniques. This review aims to provide a comprehensive overview of the current treatment options for nonunion in long bone fractures by examining both established approaches and emerging strategies.

Methods

A comprehensive literature search was conducted using PubMed, Scopus, and Google Scholar databases to identify relevant studies on fracture non-union in trauma and orthopaedics. The search was performed in October 2025 and covered publications from January 2000 to October 2025, capturing the evolution of modern non-union management principles and techniques. The following search terms and combinations were used: "fracture non-union", "fracture nonunion", "delayed union", "hypertrophic nonunion", "atrophic nonunion", "infected nonunion", "bone healing failure", "bone grafting", "revision fixation", "distraction osteogenesis", "Masquelet technique", and "intramedullary nailing".

Studies were included if they were published in English-language, peer-reviewed journals and addressed the epidemiology, pathophysiology, risk factors, classification, diagnosis, or management of fracture non-union. This included original research articles, systematic reviews, meta-analyses, clinical guidelines, case series, and relevant technical reports. Studies were excluded if they focused exclusively on spinal fractures, pathological fractures secondary to malignancy, or pediatric fracture healing without applicability to adult trauma, or if they did not provide substantive information on non-union etiology or treatment.

Initial screening was performed by reviewing titles and abstracts to identify potentially relevant articles, followed by full-text assessment of eligible studies. Reference lists of selected articles were manually searched to identify additional relevant publications. Priority was given to higher-level evidence, including systematic reviews and large prospective studies, while also incorporating established surgical techniques and emerging technologies in the field.

Given the substantial heterogeneity in study designs, anatomical locations, non-union classifications, treatment modalities, and outcome measures across the included literature, quantitative meta-analysis was not performed. Instead, data were synthesized narratively across key clinical domains, including normal fracture healing biology, non-union pathophysiology, risk factors, diagnostic approaches, non-operative management, surgical techniques, location-specific considerations, and outcomes. Where available, specific numeric data, including union rates, success percentages, complication rates, and statistical measures from individual studies, were incorporated to provide quantitative context. Consensus findings across multiple studies were emphasized, and when conflicting evidence existed, different perspectives were presented with their respective evidence base.

## Review

Normal fracture healing: understanding the baseline

To understand the pathophysiology of nonunion, we must first understand the normal cascade of fracture healing. Bone healing occurs through primary or secondary bone healing, depending on the mechanical environment and treatment approach.

Primary bone healing, also known as direct cortical healing, occurs when fracture fragments are anatomically reduced and rigidly stabilized, with minimal interfragmentary motion. This process involves direct remodeling of bone through cutting cones, where osteoclasts create tunnels across the fracture line, followed by deposition of new bone by osteoblasts. This type of healing occurs predominantly in fractures treated with compression plating and requires absolute stability with less than 2% of interfragmentary strain [6].

Secondary bone healing, the most common pathway, occurs through callus formation and involves several overlapping phases. The inflammatory phase begins immediately after fracture, with hematoma formation and release of inflammatory mediators, growth factors, and cytokines. This phase lasts several days and creates the foundation for subsequent healing. The repair phase is characterized by soft callus formation, as mesenchymal stem cells differentiate into chondrocytes and begin producing cartilage. This cartilaginous callus gradually becomes mineralized and transforms into woven bone via endochondral ossification. The remodeling phase can last years and involves the gradual transformation of the woven bone into an organized lamellar bone aligned along stress lines, ultimately restoring the bone's mechanical properties and architecture [7].

This healing process requires an adequate blood supply, an appropriate mechanical environment, viable bone and soft tissues, and the presence of necessary growth factors and cellular components. Disruption of any of these elements can impair healing and potentially lead to nonunions.

Definition and classification of nonunion

The precise definition of nonunion has been debated in orthopedic literature. The United States Food and Drug Administration (FDA) defines nonunion as a fracture that has not healed within nine months and shows no progressive signs of healing for three consecutive months. However, clinical practice often employs pragmatic definitions based on specific bone and fracture patterns. Generally, nonunion is suspected when a fracture fails to show radiographic evidence of progressive healing after approximately six to nine months for long bones [8,9].

It is important to distinguish nonunion from delayed union, which refers to slower-than-expected healing but with continued progress toward union. This distinction has important treatment implications, as delayed unions may heal with continued conservative management and monitoring, whereas true nonunions often require intervention.

Several classification systems have been proposed to categorize nonunions, with Weber-Cech classification being the most widely accepted (Table 1). This system offers a useful framework for understanding the biological basis of nonunion and helps guide treatment decisions by categorizing nonunions according to their biological activity and underlying pathophysiology [10].

**Table 1:** Classification of nonunion Table Credit: Ahmed Mohamed (author); Based on information from Solomin et al., 2023 [10]

Type	Radiographic Appearance	Pathophysiology	Causes	Treatment Principle
Hypertrophic	Abundant callus formation; "elephant foot" or "horse hoof" configuration	Adequate biology but insufficient mechanical stability; excessive motion prevents callus maturation	Inadequate fixation, hardware failure, non-compliance with weight-bearing, unstable fracture patterns	Mechanical stabilization; biological augmentation usually not needed
Atrophic	Minimal or absent callus; osteoporotic or sclerotic bone ends with polished appearance	Compromised vascularity and inadequate biological environment	Soft tissue stripping, high-energy trauma, radiation, poor blood supply (scaphoid, femoral neck), smoking, diabetes, malnutrition	Both mechanical stabilization AND biological enhancement (bone grafting)
Infected	Variable appearance depending on underlying type	Hostile environment from bacterial toxins, inflammation, biofilm formation; bacteria inhibit osteoblasts and stimulate osteoclasts	Open fractures, postoperative infections, hematogenous seeding, high-energy trauma, contaminated wounds, immunocompromised states	Staged treatment: infection control first, then definitive reconstruction

Risk factors and etiology

The development of fracture nonunion results from a complex interplay of patient-, injury-, and treatment-related factors. Understanding these risk factors is crucial for the prevention and risk stratification of fractures.

Patient-Related Factors

Patient-related factors significantly influence the fracture healing capacity. Advanced age reduces healing potential due to decreased cellular activity, reduced growth factor production, and compromised vascular response. Comorbidities, such as diabetes mellitus, hypothyroidism, and chronic kidney disease, can impair bone metabolism and healing capacity. Diabetes affects healing through multiple mechanisms, including impaired angiogenesis, reduced growth factor expression, and increased susceptibility to infection. Smoking is a significant risk factor, as nicotine causes vasoconstriction, reduces blood and oxygen delivery to tissues, and impairs cellular proliferation. Excess alcohol similarly impairs bone metabolism and interferes with calcium and vitamin D homeostasis. Nutritional status plays an important role in bone healing, as deficiencies in protein, vitamin D, calcium, and other micronutrients can impair the healing process. Certain medications, including nonsteroidal anti-inflammatory drugs (NSAIDs) and corticosteroids, have been implicated in delayed healing, although the degree of clinical significance remains debatable [11].

Injury-Related Factors

Injury-related factors include the severity of the initial trauma, vascular disruption, and bone comminution. High-energy injuries cause more extensive soft tissue and periosteal damage. Open fractures have higher nonunion rates due to soft tissue stripping, contamination, and compromised blood supply. Certain fracture patterns are more prone to nonunion, including transverse fractures with less surface contact area, segmental fractures with a compromised blood supply to the intermediate segment, and fractures with significant bone loss. Anatomical location significantly influences healing potential, with certain bones having inherently poor blood supply or being subjected to greater mechanical demands. The scaphoid bone, ulna, femoral neck, tibial shaft, and fifth metatarsal (Jones fracture) are susceptible to nonunion due to their vascular anatomy and mechanical environment [8,12].

Treatment-Related Factors

Treatment-related factors include the surgical technique, implant selection, and postoperative management. Inadequate reduction, which leaves gaps at the fracture site, impairs healing by increasing the distance that must be bridged by new bone formation. Poor fixation stability allows excessive motion at the fracture site and prevents consolidation during the early phases of healing. Conversely, excessive rigidity in some situations may impair secondary bone healing by preventing beneficial micromotion that stimulates callus formation. Surgical approaches that extensively strip the periosteum and soft tissues compromise the blood supply and devitalize bone fragments. Infection remains a significant complication that can completely arrest healing, with the presence of bacteria, inflammatory mediators, and necrotic tissue creating an environment hostile to bone regeneration [13].

Pathophysiology of nonunion development

The development of fracture nonunion involves the disruption of the normal healing cascade through mechanical, biological, and infective mechanisms. Understanding these pathophysiological processes is essential for targeted treatment approaches.

Mechanical Factors

Mechanical stability at the fracture site is crucial for bone healing. Excessive motion at the fracture site, termed "micromotion," within physiological limits, can stimulate callus formation. However, excessive instability prevents progression from cartilaginous to bony callus, resulting in hypertrophic nonunion. The fracture gap also plays a critical role; large gaps require large callus formation and are prone to nonunion, particularly in the presence of instability [14].

Biomechanical forces at the fracture site vary by anatomical location and directly influence healing and fixation strategy. Weight-bearing bones, such as the femur and tibia, are subjected mainly to compressive and bending forces during ambulation; controlled compression promotes callus formation, whereas excessive shear or bending disrupts healing and predisposes to nonunion. In contrast, forearm fractures experience significant rotational stresses from pronation and supination, necessitating rigid fixation and precise anatomical alignment to restore rotational stability. Clavicular and humeral fractures encounter combined bending and torsional loads from upper limb motion, requiring fixation that resists these multidirectional forces. Therefore, understanding the unique loading environment and strain characteristics of each bone is crucial for selecting fixation methods that provide adequate stability while preserving the micromotion necessary for secondary bone healing [15].

Biological Factors

The biological environment at the fracture site largely determines the healing capacity. The periosteum, a thin but highly vascular membrane surrounding the bone, plays a vital role in bone healing by supplying osteoprogenitor cells, growth factors, and blood vessels essential for new bone formation. Therefore, the preservation of the periosteum during surgery is important. Excessive periosteal stripping and improper soft tissue handling during surgery or injury associated with excessive soft tissue damage significantly reduce the healing potential and increase the risk of nonunion [16].

Certain bones, as mentioned before, possess inherently poor blood supply, making them particularly vulnerable. At the cellular level, fracture repair depends on a coordinated cascade of growth factors, cytokines, and signaling pathways that regulate cellular differentiation and matrix production. Disruption of these processes through injury, infection, or systemic disease can impair healing by limiting the availability and function of stem cells at the fracture site [17].

Infection

Infection of the fracture site creates a hostile environment for bone healing. Bacterial colonization, particularly biofilm formation on implants, triggers chronic inflammation that inhibits osteogenesis. Infected nonunions require addressing both infection and mechanical/biological factors preventing union, often necessitating staged treatment protocols [18].

Diagnosis and evaluation

Accurate diagnosis and comprehensive evaluation are prerequisites for successful nonunion management. The diagnostic process combines clinical, radiographic, and biochemical assessments.

Clinical Assessment

Clinical assessment begins with a history taking. This should cover the initial injury, treatment methods, progression of healing, and current functional limitations. Determining time since injury helps to distinguish a delayed union from a true nonunion. A review of previous interventions and their outcomes provides insights into potential treatment challenges. Additionally, a comprehensive evaluation of patient-related factors, such as smoking, medications, nutrition, and comorbidities, is crucial for understanding the biological environment of fractures. 

The next step is clinical examination. Patients with nonunion present with persistent pain, often exacerbated by weight-bearing or use, and instability at the fracture site. A detailed neurovascular assessment should be performed.

Radiographic Assessment

Plain radiography remains the primary imaging modality for nonunion diagnosis and classification. Multiple views should be obtained to assess all aspects of the nonunion. Radiographic signs of nonunion include persistent fracture lines, lack of bridging callus, sclerosis of fracture ends, rounding or atrophy of bone ends, and the presence of a visible gap. Serial radiographs demonstrating an absence of progressive healing over a three- to six-month period are indicative of a fracture progressing towards nonunion.

Computed tomography (CT) provides detailed cross-sectional imaging that enhances the evaluation of fracture healing, particularly when radiographic findings are inconclusive. CT allows precise assessment of cortical continuity and can demonstrate bridging calluses across the fracture site, confirming union when present. CT is especially valuable in complex or obliquely oriented fractures or when metallic implants obscure radiographic visualization. However, CT may underestimate healing in the early stages, as immature or fibrous calli may not yet be sufficiently mineralized to appear radiodense. In addition, CT aids in the identification of bone defects, assessment of implant integrity, and planning of revision surgery [19].

Magnetic resonance imaging (MRI) provides superior soft tissue contrast and valuable information about the biological activity of a nonunion. It enables a detailed assessment of bone marrow edema patterns, which may indicate ongoing biological activity or persistent stress at the fracture site. MRI can evaluate the vascularity of the bone ends, an essential factor in distinguishing viable from avascular segments, and in planning procedures such as vascularized bone grafting. Its ability to visualize the surrounding soft tissues also allows the detection of fibrous tissue interposition, synovial pseudarthrosis, or fluid collections that may interfere with healing. Importantly, MRI is highly sensitive in identifying occult infections, demonstrating features such as marrow signal changes, sinus tracts, and soft-tissue abscesses that may not be evident on CT or radiography. Metal artifact reduction sequences (MARS) further enhance diagnostic utility in patients with fixation devices. Overall, MRI complements CT by providing crucial insights into the biological and soft tissue environment of nonunion, guiding both diagnosis and surgical planning [1,20].

Nuclear medicine studies, including bone scintigraphy and positron emission tomography (PET) scans, demonstrate metabolic activity at the fracture site. These studies can help distinguish between hypertrophic and atrophic nonunions based on metabolic activity patterns and may aid in detecting infection when combined with labeled white blood cell scans [21].

Biochemical Assessment

Laboratory assessment plays an important role in the evaluation of nonunion, particularly in identifying underlying infection, metabolic abnormalities, or systemic factors that may impair healing. Inflammatory markers, such as erythrocyte sedimentation rate (ESR), C-reactive protein (CRP), and white cell count (WCC), are routinely used when infection is suspected; however, they lack specificity and should be interpreted in conjunction with clinical and imaging findings. Full blood count (FBC) may reveal leukocytosis in acute infections but is often normal in chronic low-grade cases [22,23].

Evaluation of the patient’s nutritional and metabolic status is equally important, as deficiencies can directly influence bone regeneration. Measurement of serum albumin, vitamin D, calcium, and phosphate levels helps identify correctable deficits that may compromise healing. In patients with delayed or atypical healing, parathyroid hormone, thyroid function, and renal profile tests may be warranted to exclude metabolic bone disease or endocrine dysfunction [24].

When an infection is strongly suspected, bone biopsy remains the gold standard for diagnosis. Multiple tissue samples should be obtained intraoperatively or percutaneously and sent for aerobic, anaerobic, and extended cultures, as well as histopathological examination, when necessary [25].

Bone grafting techniques

Bone grafting addresses biological deficiencies by providing cells, growth factors, and scaffolds for new bone formation. The choice between autograft, allograft, bone graft substitutes, and vascularized grafts depends on the size of the bone defect, vascularity of the nonunion site, availability of donor sites, and patient factors.

Autografts remain the gold standard, providing osteogenesis through living cells, osteoinduction through growth factors, and osteoconduction as scaffolds. The iliac crest is the most common donor site for harvesting bone grafts. It contains substantial quantities of cancellous and corticocancellous bones. Cancellous bone has excellent biological properties, whereas cortical bone provides good structural support. The Reamer-Irrigator-Aspirator system offers an alternative, harvesting autografts from the medullary canal during reaming procedures and avoiding a separate donor site incision [26].

Vascularized bone transfer is another advanced form of autograft that requires high expertise and specific complex cases. It is reserved for nonunions with significant bone loss, poor vascularity, or after multiple failed procedures. This technique transfers bone with an intact blood supply via microvascular anastomosis, providing both structural support and biological enhancement. The fibula is the most commonly used, providing substantial length and adequate strength. The surgical technique is complex and requires both orthopedic and microvascular expertise. Indications include large segmental defects, avascular nonunions, radiation-induced nonunions, and salvage procedures. Success rates exceed 80% in experienced hands, although the complexity limits its widespread application [27-29].

Allograft bone provides osteoconductive and osteoinductive properties, but lacks living cells, making it less potent than autografts. However, it avoids donor site morbidity and allows the use of large structural grafts. Allografts are commonly used as extenders combined with autografts or in large reconstructions requiring structural support, as in acetabular reconstruction [30].

Synthetic bone graft substitutes offer unlimited availability without risk of disease transmission. Calcium-based materials provide osteoconductive scaffolds; however, they generally do not match autograft performance in challenging nonunion cases requiring robust biological stimulation [31].

Non-operative management

Non-operative management of nonunion, although less commonly successful than surgical intervention, plays an important role in selected cases and as an adjunct to operative treatment.

Optimization of Patient Factors

Addressing modifiable risk factors is fundamental in any treatment plan. Smoking cessation is the most important intervention. Studies have demonstrated significantly improved healing rates in patients who quit smoking. Patients should be counseled extensively about the detrimental effects of smoking and offered smoking cessation resources [32,33].

Nutritional optimization involves ensuring adequate protein intake, vitamin D supplementation to achieve therapeutic levels, and calcium supplementation when the dietary intake is insufficient. Patients with significant nutritional deficiencies may benefit from nutritionist consultations. In severe cases, correction of nutritional deficits before surgical intervention may be necessary [34].

Management of comorbid conditions, including diabetes mellitus, with optimization of glycemic control and treatment of peripheral vascular disease can improve healing potential. Medication review should identify medications that affect bone and wound healing, such as rheumatoid arthritis medications. NSAIDs should be discontinued, if possible, during the active healing phase.

Functional Bracing and Protected Weight-Bearing

In selected cases of delayed union or early nonunion, continued adequate immobilization with functional bracing combined with protected weight bearing may allow healing. This approach is most applicable to hypertrophic nonunions, where biological potential exists but mechanical stability is inadequate. The principle involves providing external support while allowing physiological loading to stimulate healing [35].

However, prolonged nonoperative treatment of established nonunion often delays definitive management without achieving union. It allows for muscle atrophy, joint stiffness, and bone loss. The decision to pursue continued non-operative management must be carefully individualized.

Biophysical Stimulation

Biophysical modalities aim to enhance bone healing by stimulating cellular and molecular processes at fracture sites. Low-intensity pulsed ultrasound (LIPUS), delivered through devices such as the EXOGEN Ultrasound Bone Healing System (Bioventus LLC, Durham, North Carolina, United States), transmits mechanical energy that promotes cellular proliferation, angiogenesis, and osteogenic differentiation. Clinical studies have shown potential benefit in fresh fractures and delayed unions, though the evidence for established nonunions remains inconsistent [36,37].

Electrical stimulation techniques, including direct current stimulation, capacitive coupling, and pulsed electromagnetic fields (PEMF), modulate cell membrane potentials and calcium ion flux, enhancing osteoblast activity and matrix production. While some trials have reported improved union rates, others have demonstrated limited or no significant benefits, leading to ongoing debate regarding their efficacy [38].

These modalities are noninvasive and low-risk, making them suitable adjuncts in selected cases, particularly for patients who are poor surgical candidates or decline operative intervention. However, surgical management should not be delayed in cases of clear mechanical instability or biological deficiencies requiring operative correction.

Operative management

Operative intervention represents the mainstay of nonunion treatment, with success rates exceeding 80-90% in most series when appropriate techniques are employed [39]. The main principle of treating nonunions is to understand their cause. Other principles include restoring mechanical stability, enhancing the biological environment, and correcting associated deformities. The surgical approach varies depending on whether the nonunion follows conservative treatment or previous surgical intervention, and the specific techniques employed must address the underlying pathophysiology [40].

Treatment of Nonunions Following Conservative Management

A nonunion that develops after conservative treatment presents without hardware complications and may offer more straightforward surgical options. These cases require initial surgical stabilization combined with biological enhancement, depending on the nonunion type.

Mechanical stability is the primary requirement for hypertrophic nonunion following conservative treatment. Intramedullary nailing is preferred for diaphyseal nonunions of long bones, such as the femur, tibia, and humerus. Modern locked intramedullary nails provide excellent stability while preserving the blood supply. The reaming process facilitates the release of growth factors and generates autogenous bone graft material at the site of nonunion. Plate fixation remains a valuable option for metaphyseal and periarticular nonunions, where intramedullary devices may be less suitable. Advances in locking plate technology have further enhanced stability, even in osteoporotic bone [40].

For atrophic nonunion following conservative treatment, both mechanical stability and biological augmentation are necessary. The surgical approach combines rigid fixation with bone grafting to address the inadequate biological environment. The choice of fixation follows the same principles as hypertrophic nonunion, but biological enhancement through autogenous bone grafting is essential for success [41].

Revision Internal Fixation for Previously Operated Nonunions

Revision fixation addresses nonunions that develop despite previous surgical treatment. However, this approach is also challenging. The challenges include hardware removal, compromised soft tissues, bone loss, and the need for bone grafting. Hardware removal must be complete because retained broken screws or loose plates can compromise new fixation and serve as stress risers. The nonunion site is sufficiently debrided, removing interposed fibrous tissue while carefully preserving viable bone and soft tissue attachments that maintain the blood supply. Correction of malalignment is essential, as deformities create abnormal stresses that impede healing, even with stable fixation [42,43].

Exchange nailing is an effective technique for diaphyseal nonunion that was previously treated with intramedullary nailing. The previous nail is removed and replaced with a larger diameter nail. This approach combines mechanical stability with biological stimulation, as the reaming process releases growth factors and bone marrow contents. Success rates of 85-95% have been reported for hypertrophic diaphyseal nonunions treated with exchange nailing [44,45].

This revision may involve more robust plating techniques for nonunions previously treated with plate fixation. Dual plating, in which two plates are placed at different positions around the bone circumference, provides enhanced stability in complex cases. In some situations, conversion from plate fixation to intramedullary nailing or vice versa may be appropriate, depending on the specific anatomy and failure pattern [46,47].

External fixation plays an important role when internal fixation fails or is unadvisable. In infected nonunions, external fixators provide temporary or definitive stabilization while avoiding placement of internal implants in contaminated tissue. Circular external fixators, particularly the Ilizarov apparatus, enable gradual correction of deformities, limb lengthening, and definitive treatment of complex nonunions [48,49].

Management of Infected Nonunion

Infected nonunion requires special consideration irrespective of prior interventions. The fundamental principle of management is eradication or control of infection prior to, or in conjunction with, definitive reconstruction. Staged treatment is necessary, with the first stage involving debridement of all infected tissues, removal of hardware when possible, obtaining tissue cultures, and temporary stabilization with external fixation. Antibiotic therapy based on culture results is initiated. After infection control is achieved, definitive reconstruction is performed with revision fixation and bone grafting [50].

Antibiotic therapy involves initial broad-spectrum coverage, followed by organism-specific antibiotics once cultures identify pathogens and sensitivities. Treatment requires prolonged courses of intravenous antibiotics, often six weeks or longer, followed by oral suppression in some cases. Antibiotic beads or spacers placed at the surgical site provide high local concentrations of antibiotics while minimizing systemic toxicity. These polymethylmethacrylate (PMMA) beads impregnated with antibiotics gradually elute drugs over weeks, sterilizing the local environment [51].

The masquelet-induced membrane technique offers an alternative for infected nonunions with bone loss. Thorough debridement creates a defect that is filled with an antibiotic-impregnated cement spacer. Over weeks to months, a biological membrane forms around the spacer. In the second stage, the spacer is removed, and the induced membrane is filled with a cancellous autograft, with high success rates reported. In selected cases with early infection and less virulent organisms, single-stage treatment combining debridement, antibiotic therapy, and definitive fixation can be successful [52,53].

Outcomes and prognosis

The prognosis for nonunion treatment varies considerably based on multiple factors, including the anatomical location, nonunion type, presence of infection, extent of bone loss, patient comorbidities, and treatment method employed. Understanding these variables helps set realistic expectations and guide treatment decisions.

Hypertrophic nonunion generally has a favorable prognosis because the biological environment for healing remains active. Abundant callus formation indicates good vascularity and cellular activity, suggesting that the main problem lies in inadequate stability rather than poor biology. Once proper mechanical fixation and alignment are restored, these fractures unite well without the need for additional biological stimulation. Overall, with appropriate stabilization, the outcome is excellent, and union is usually achieved within a predictable timeframe [54].

Atrophic nonunions present greater challenges because of biological failure requiring augmentation. However, with appropriate treatment, including stable fixation and bone grafting, union rates of 80-90% are achievable in many locations. Multiple procedures may be necessary, and the treatment duration is longer than that for hypertrophic nonunions [41,55].

Infected nonunions have a more guarded prognosis, with success dependent on completely eradicating the infection while simultaneously achieving bony union. Even with optimal treatment, these patients may require multiple surgeries, prolonged antibiotic courses, and extended treatment timelines lasting a year or more. Success rates vary widely depending on the severity of infection, extent of bone loss, and patient factors, but range from 60% to 80% for union with infection eradication in specialized centers. Some patients require amputation when reconstruction is impossible or creates more morbidity than amputation [56,57].

Nonunions with significant bone loss have outcomes tied to the reconstruction method employed. The Masquelet technique reports union rates of 70-90% for defects up to 8 cm, although multiple procedures are often necessary. Bone transport achieves similar success rates but requires prolonged external fixation time, and complications, including pin site infections, wire breakage, and premature consolidation, are common. Vascularized bone grafts achieve high union rates but require specialized microsurgical expertise and may develop complications, including graft fracture, nonunion at the graft-host junctions, or donor site morbidity [58-61].

Functional outcomes depend not only on achieving bony union but also on restoring alignment, length, and preventing complications. Many patients with nonunion experience prolonged disability even after successful treatment due to muscle atrophy, joint stiffness, post-traumatic arthritis, or residual deformity. Return to work rates vary, with physically demanding occupations presenting greater challenges. Quality of life assessments in patients with nonunion demonstrate significant impacts on physical function, mental health, and social well-being that may persist despite achieving union [62-64].

Complications of nonunion treatment include infection, hardware failure, nerve injury, malunion, leg length discrepancy, and failure to achieve union, requiring further surgery. The risk of complications increases with surgical complexity, with procedures involving extensive soft tissue dissection, bone grafting, or external fixation carrying higher rates of problems. Managing patient expectations regarding treatment duration, number of procedures, and functional outcomes is essential for satisfaction.

Future directions and emerging technologies

The field of nonunion treatment continues to evolve with advances in biological augmentation, fixation technology, and regenerative medicine approaches. Several emerging areas show promise in improving outcomes.

Cell-based therapies, including mesenchymal stem cells, bone marrow aspirate concentrate, and platelet-rich plasma, aim to provide the cellular and growth factor components that are necessary for healing. Although early results are encouraging, clinical evidence remains limited, and questions regarding optimal cell sources, preparation methods, dosing, and delivery timing require further investigation. These therapies may eventually provide off-the-shelf biological augmentation without requiring autograft harvesting [65,66].

Advanced biomaterials continue to evolve with new synthetic bone graft substitutes incorporating growth factors, improved osteoconductive properties, and controlled degradation profiles. Three-dimensionally printed scaffolds customized to individual defect geometries may improve the reconstruction of complex bone loss. Bioactive materials that actively promote cellular attachment, proliferation, and differentiation are an area of active investigation [67,68].

Regenerative rehabilitation, which integrates physical therapy principles with biological healing phases, aims to optimize mechanical stimulation during healing, while avoiding excessive loading that could impair consolidation [69]. Evidence-based protocols for progressive weight-bearing and functional loading may improve outcomes and accelerate return to function.

## Conclusions

Nonunion following long bone fractures remains a challenging complication requiring comprehensive evaluation and individualized treatment. Successful management depends on addressing both mechanical instability and biological deficiencies, while considering patient factors, anatomical location, and previous treatments. Operative intervention provides the highest success rates, with modern techniques achieving union in a high percentage of cases through combinations of revision fixation, bone grafting, and biological augmentation. Hypertrophic nonunions respond primarily to improved mechanical stability, while atrophic nonunions require biological enhancement. Infected nonunions necessitate staged treatment, prioritizing infection control.

Advanced techniques, including distraction osteogenesis and vascularized bone grafts, offer solutions for complex cases with bone loss or poor vascularity. Patient factors, particularly smoking cessation and nutritional optimization, significantly influence outcomes. Emerging technologies, including advanced biologics, three-dimensional printing, and novel fixation devices, promise continued improvement in nonunion management. Optimal initial fracture treatment, early recognition of healing problems, and timely intervention when nonunion develops remain paramount to minimise the adverse impact of nonunion on patient outcomes.

## References

[1] Ghanem W, Ezzeddine H, Saad R (2025). State of the nonunion: a review of the latest literature. Orthop Rev (Pavia).

[2] Steinmann SP, Adams JE (2006). Scaphoid fractures and nonunions: diagnosis and treatment. J Orthop Sci.

[3] Flores MJ, Brown KE, O'Marr JM (2024). The economic impact of infection and/or nonunion on long-bone shaft fractures: a systematic review. OTA Int.

[4] Crawford RR (1973). A history of the treatment of non-union of fractures in the 19th century, in the United States. J Bone Joint Surg Am.

[5] Simonis RB (2000). An historical background to the treatment of non-union. Orthofix External Fixation in Trauma and Orthopaedics.

[6] Bigham-Sadegh A, Oryan A (2015). Basic concepts regarding fracture healing and the current options and future directions in managing bone fractures. Int Wound J.

[7] Marsell R, Einhorn TA (2011). The biology of fracture healing. Injury.

[8] Gharu E, John B (2024). Nonunion of fractures: a review of epidemiology, diagnosis, and clinical features in recent literature. Indian J Orthop.

[9] Wittauer M, Burch MA, McNally M (2021). Definition of long-bone nonunion: a scoping review of prospective clinical trials to evaluate current practice. Injury.

[10] Solomin LN, Semenistyy AA, Komarov AV, Khominets VV, Sheridan GA, Rozbruch SR (2023). Universal long bone nonunion classification. Strategies Trauma Limb Reconstr.

[11] Hernandez RK, Do TP, Critchlow CW, Dent RE, Jick SS (2012). Patient-related risk factors for fracture-healing complications in the United Kingdom General Practice Research Database. Acta Orthop.

[12] Nicholson JA, Makaram N, Simpson A, Keating JF (2021). Fracture nonunion in long bones: a literature review of risk factors and surgical management. Injury.

[13] Mills L, Tsang J, Hopper G, Keenan G, Simpson AH (2016). The multifactorial aetiology of fracture nonunion and the importance of searching for latent infection. Bone Joint Res.

[14] Yang Q, Xu M, Fang H, Gao Y, Zhu D, Wang J, Chen Y (2024). Targeting micromotion for mimicking natural bone healing by using NIPAM/Nb(2)C hydrogel. Bioact Mater.

[15] Mehboob A, Barsoum I, Al-Rub RK (2025). Review on lower limb bone fracture fixation: biomechanics, design, and advancements in additive manufacturing for implants. Mater Des.

[16] Li C (2023). Correspondence: perspectives on the future of dysmorphology. Am J Med Genet A.

[17] Schell H, Duda GN, Peters A, Tsitsilonis S, Johnson KA, Schmidt-Bleek K (2017). The haematoma and its role in bone healing. J Exp Orthop.

[18] Bose D, Kugan R, Stubbs D, McNally M (2015). Management of infected nonunion of the long bones by a multidisciplinary team. Bone Joint J.

[19] Nicholson JA, Yapp LZ, Keating JF, Simpson AH (2021). Monitoring of fracture healing. Update on current and future imaging modalities to predict union. Injury.

[20] McNally EG, Goodman R, Burge P (2000). The role of MRI in the assessment of scaphoid fracture healing: a pilot study. Eur Radiol.

[21] Gandhi SJ, Rabadiya B (2017). Bone scan in detection of biological activity in nonhypertrophic fracture nonunion. Indian J Nucl Med.

[22] Shapiro JA, Stillwagon MR, Tornetta P 3rd (2022). Serology and comorbidities in patients with fracture nonunion: a multicenter evaluation of 640 patients. J Am Acad Orthop Surg.

[23] Liu T, Li F, Yan Y (2025). The role of persistent inflammation in the pathogenesis of infected nonunion: a narrative review. Immun Inflamm Dis.

[24] Christianson E, Thomas M, Sprague S, Rivera J, Chapple A, Zura R (2024). Nutritional indicators of bone nonunion: a systematic review. J Clin Med.

[25] Sousa R, Carvalho A, Santos AC, Abreu MA (2021). Optimal microbiological sampling for the diagnosis of osteoarticular infection. EFORT Open Rev.

[26] Peterson JR, Chen F, Nwankwo E, Dekker TJ, Adams SB (2019). The use of bone grafts, bone graft substitutes, and orthobiologics for osseous healing in foot and ankle surgery. Foot Ankle Orthop.

[27] Adaş M (2024). Treatment of AVN-induced proximal pole scaphoid nonunion using a fifth and fourth extensor compartmental artery as a vascularized pedicle bone graft: a retrospective case series. Med Sci Monit.

[28] Karimnazhand R, Shams R, Behmanesh A (2025). Comparative efficacy of non-vascularized and vascularized bone grafts, with emerging insights into bone biomaterial grafts, in scaphoid fracture nonunion treatment: a systematic review and meta-analysis. J Orthop Translat.

[29] Rizzo M, Moran SL (2008). Vascularized bone grafts and their applications in the treatment of carpal pathology. Semin Plast Surg.

[30] Movaniya P, Chhatbar R, Bhatt S, Sing NJ, Arya A, Parmar H (2025). Comparison of osteoinductive potential of autografts vs. Allografts in mandibular defect models. J Pharm Bioallied Sci.

[31] Ibrahim M, Navaneethakrishnapandian M, Kumanan M (2024). Effect of synthetic bone graft substitutes in management of nonunion in long bones: a case series. J Orthop Joint Surg.

[32] Lu Y, Di YP, Chang M (2021). Cigarette smoke-associated inflammation impairs bone remodeling through NFκB activation. J Transl Med.

[33] Hernigou J, Schuind F (2019). Tobacco and bone fractures: a review of the facts and issues that every orthopaedic surgeon should know. Bone Joint Res.

[34] Karpouzos A, Diamantis E, Farmaki P, Savvanis S, Troupis T (2017). Nutritional aspects of bone health and fracture healing. J Osteoporos.

[35] Bowers KM, Anderson DE (2024). Delayed union and nonunion: current concepts, prevention, and correction: a review. Bioengineering (Basel).

[36] Higgins A, Glover M, Yang Y, Bayliss S, Meads C, Lord J (2014). EXOGEN ultrasound bone healing system for long bone fractures with non-union or delayed healing: a NICE medical technology guidance. Appl Health Econ Health Policy.

[37] Palanisamy P, Alam M, Li S, Chow SK, Zheng YP (2022). Low-intensity pulsed ultrasound stimulation for bone fractures healing: a review. J Ultrasound Med.

[38] Farjaminejad S, Dingle AM (2025). The role of electrical stimulation in bone regeneration: mechanistic insights and therapeutic advances. Bioelectron Med.

[39] Phillips MR, Harrison A, Mehta S, Nolte PA, Bhandari M, Zura R (2022). A scoping review of operative and non-invasive management in the treatment of non-unions. Injury.

[40] Marongiu G, Dolci A, Verona M, Capone A (2020). The biology and treatment of acute long-bones diaphyseal fractures: overview of the current options for bone healing enhancement. Bone Rep.

[41] Sun L, Li Z, Ma T (2019). Treatment of atrophic nonunion via autogenous ilium grafting assisted by vertical fixation of double plates: a case series of patients. J Int Med Res.

[42] Ziranu A, Noia G, Cipolloni V (2022). Revision surgery using retrograde nail versus replating in nonunion distal femur fracture treated with plate. Adv Orthop.

[43] Ali A, Douglas H, Stanley D (2005). Revision surgery for nonunion after early failure of fixation of fractures of the distal humerus. J Bone Joint Surg Br.

[44] Brinker MR, O'Connor DP (2007). Exchange nailing of ununited fractures. J Bone Joint Surg Am.

[45] Gálvez-Sirvent E, Ibarzábal-Gil A, Rodríguez-Merchán EC (2020). Treatment options for aseptic tibial diaphyseal nonunion: a review of selected studies. EFORT Open Rev.

[46] Barwar N, Gargi G, Rai A, Elhence A, Banerjee S, Gahlot N (2025). Dual plating in the management of nonunion complex distal femur fractures following lateral locked plate fixation: radiological and functional outcomes of a prospective study. J Trauma Inj.

[47] Peng Y, Ji X, Zhang L, Tang P (2016). Double locking plate fixation for femoral shaft nonunion. Eur J Orthop Surg Traumatol.

[48] Simpson AH, Robiati L, Jalal MM, Tsang ST (2019). Non-union: Indications for external fixation. Injury.

[49] Fahad S, Habib AA, Awais MB, Umer M, Rashid HU (2019). Infected non-union of tibia treated with Ilizarov external fixator: our experience. Malays Orthop J.

[50] Gómez-Barrena E, Ehrnthaller C (2024). Long bone uninfected non-union: grafting techniques. EFORT Open Rev.

[51] Masrouha KZ, Raad ME, Saghieh SS (2018). A novel treatment approach to infected nonunion of long bones without systemic antibiotics. Strategies Trauma Limb Reconstr.

[52] Ahmed H, Shakshak M, Trompeter A (2025). A review of the Masquelet technique in the treatment of lower limb critical-size bone defects. Ann R Coll Surg Engl.

[53] Chloros GD, Kanakaris NK, Harwood PJ, Giannoudis PV (2022). Induced membrane technique for acute bone loss and nonunion management of the tibia. OTA Int.

[54] Uzun M, Kara A, Bülbül AM, Çakar M (2015). Treatment of aseptic hypertrophic nonunion of the lower extremity with less invasive stabilization system (new approach to hypertrophic nonunion treatment). Adv Orthop Surg.

[55] Ehrnthaller C, Hoxhaj K, Manz K, Zhang Y, Fürmetz J, Böcker W, Linhart C (2024). Preventing atrophic long-bone nonunion: retrospective analysis at a level I trauma center. J Clin Med.

[56] Bauer T, Klouche S, Grimaud O, Lortat-Jacob A, Hardy P (2018). Treatment of infected non-unions of the femur and tibia in a French referral center for complex bone and joint infections: outcomes of 55 patients after 2 to 11 years. Orthop Traumatol Surg Res.

[57] Marais LC, Zalavras CG, Moriarty FT, Kühl R, Metsemakers WJ, Morgenstern M (2024). The surgical management of fracture-related infection. Surgical strategy selection and the need for early surgical intervention. J Orthop.

[58] Durand M, Mathieu L, Venant J, Masquelet AC, Collombet JM (2025). Engineering the bone reconstruction surgery: the case of the masquelet-induced membrane technique. Eur J Trauma Emerg Surg.

[59] Mi M, Papakostidis C, Wu X, Giannoudis PV (2020). Mixed results with the Masquelet technique: a fact or a myth?. Injury.

[60] Shastov AL, Kolchin SN, Malkova TA (2025). Effectiveness of the induced membrane technique in aseptic and infected long-bone defect management: are there any differences?. World J Orthop.

[61] Alford AI, Nicolaou D, Hake M, McBride-Gagyi S (2021). Masquelet's induced membrane technique: review of current concepts and future directions. J Orthop Res.

[62] Barker KL, Lamb SE, Simpson AH (2004). Functional recovery in patients with nonunion treated with the Ilizarov technique. J Bone Joint Surg Br.

[63] Wagner RK, Emmelot MP, Ly TV, Harris MB, Janssen SJ, Kloen P (2024). Long-term patient reported outcomes after revision surgery for lower extremity nonunion: a retrospective cohort study. Injury.

[64] Boadi BI, Konda SR, Denasty A, Leucht P, Egol KA (2023). No decay in outcomes at a mean 8 years following surgical treatment for aseptic fracture nonunion. Injury.

[65] Kiwan E, Ghanem W, Ezzeddine H (2025). Revolutionizing nonunion treatment: the expanding role of local biological therapies. Orthop Rev (Pavia).

[66] Cui C, Lin F, Xia L, Zhang X (2025). Mesenchymal stem cells therapy for the treatment of non-union fractures: a systematic review and meta-analysis. BMC Musculoskelet Disord.

[67] Sun J, Chen C, Zhang B, Yao C, Zhang Y (2025). Advances in 3D-printed scaffold technologies for bone defect repair: materials, biomechanics, and clinical prospects. Biomed Eng Online.

[68] Turnbull G, Clarke J, Picard F (2018). 3D bioactive composite scaffolds for bone tissue engineering. Bioact Mater.

[69] Deng C, Aldali F, Luo H, Chen H (2024). Regenerative rehabilitation: a novel multidisciplinary field to maximize patient outcomes. Med Rev (2021).

